# Real-Space Image of Charged Patches in Tunable-Size Nanocrystals

**DOI:** 10.3390/ma15041455

**Published:** 2022-02-15

**Authors:** Jordi Martínez-Esaín, Ana Pérez-Rodríguez, Jordi Faraudo, Esther Barrena, Ramón Yáñez, Carmen Ocal, Susagna Ricart

**Affiliations:** 1 Departament de Química, Universitat Autònoma de Barcelona (UAB), 08193 Barcelona, Spain; martinezesain.jordi@protonmail.com (J.M.-E.); ramon.yanez@uab.cat (R.Y.); 2 Institut de Ciència de Materials de Barcelona, ICMAB-CSI C, Campus de la UAB, 08193 Barcelona, Spain; ana.perez.rodriguez.c@gmail.com (A.P.-R.); ebarrena@icmab.es (E.B.); cocal@icmab.es (C.O.)

**Keywords:** patchy nanocrystals, charged patches, HRTEM, KPFM

## Abstract

The remarkable dual nature of faceted-charge patchy metal fluoride nanocrystals arises from the spontaneous selective coordination of anionic and cationic ligands on the different facets of the nanocrystals. In previous studies, the identification and origin of the charge at the patches were obtained by combining computer simulations with indirect experimental evidence. Taking a step further, we report herein the first direct real-space identification by Kelvin probe force microscopy of the predicted faceted-charge patchy behavior, allowing the image of the dual faceted-charge surfaces. High-resolution transmission electron microscopy reveals the detailed nanocrystal faceting and allows unambiguously inferring the hydrophilic or hydrophobic role of each facet from the identification of the surface atoms exposed at the respective crystallographic planes. The success of the study lies in a foresighted synthesis methodology designed to tune the nanocrystal size to be suitable for microscopy studies and demanding applications.

## 1. Introduction

**Figure 1:**
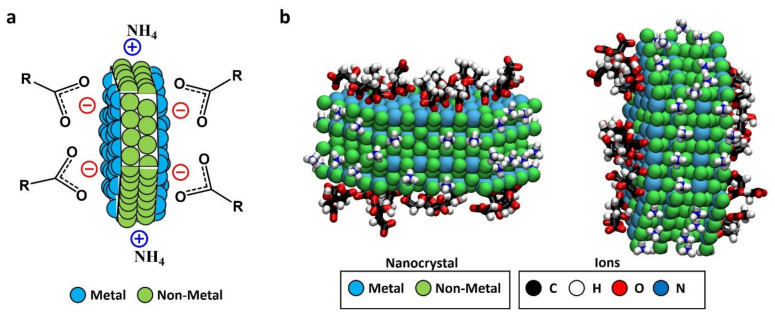
(**a**) Schematic representation of faceted-charge patchy NC with the local charges in the exposed patches due to the ionic stabilizers; (**b**) snapshot (two different views) generated from the results of our previous MD simulations [7] of a LaF_3_ NC in solution (NC atoms are shown as van der Waals spheres, and adsorbed ions are shown in licorice representation; water and non-adsorbed ions are not shown for simplicity). The images show adsorbed acetate and citrate anions (found only at the hexagonal face) and adsorbed ammonium cations (found only at the rectangular face), thereby predicting the formation of charged patches due to ligand adsorption. These adsorptions gave a charge density of approximately −3.4 e/nm^2^ for hexagonal facets and +3.4 e/nm^2^ for the rectangular ones, obtaining a global charge density of −0.47 e/nm^2^ considering the NC. Images in (**b**) were generated with Visual Molecular Dynamics software (VMD) version 1.9.3 (Urbana—Champaign, IL, USA) [10].

Currently, there is substantial activity on the development and fabrication of particles with nanopatterned surfaces, i.e., particles with chemically or topographically distinct surface domains (patches) [1]. One of the reasons for this interest is their use as building blocks in the self-assembly of complex hierarchical structures, with the patches providing localized interaction sites promoting self-assembly [2,3,4,5,6]. Efficient methods for the generation of micrometer -and sub-micrometer-sized patchy colloids have already been developed. Currently, the efforts are directed toward the search for patchy nanocrystals (NCs), with sizes of the order of tens of nanometers [1,2]. For example, Choueiri et al. obtained discrete patches of various types of polymers over gold NCs [1]. The origin of the patches in that case was the thermodynamic segregation of the different polymers. Recently, we obtained patchy LnF_3_ NCs (Ln: La, Ce, or Pr) with patches exhibiting different electric charges [7]. As shown schematically in Figure 1a, the charge duality arises from the selective functionalization of the diverse NC patches with anionic and cationic ligands. The underlying mechanism, identified by molecular dynamics simulations, is due to the spontaneous faceting of hexagonal LnF_3_ NCs, which leads to planes with different affinities for different kinds of ligands. LnF_3_ NCs expose Ln metal ions at their hexagonal planes and F atoms at their rectangular planes. In general, ligands containing carboxylate moieties coordinate with metal atoms (X-type coordination following the covalent bond classification for nanoscale systems) [8,9], while nitrogen-based molecules interact with fluorine atoms (electrostatic interaction based on H-bonding (F···H–N). The hexagonal surfaces of LnF_3_ NCs are stabilized by citrate and acetate anions coordinated to Ln, acquiring a negative charge, and the rectangular surfaces are stabilized by ammonium cations, acquiring a positive charge. This effect is illustrated in Figure 1b as seen in MD simulations. Experimentally, we confirmed the presence of adsorbed ligands (citrate, acetate, and ammonium) in LnF_3_ NCs by combining ^1^H-NMR and IR characterization. Therefore, each facet results spontaneously functionalized and charged in a selective way by the ligands present during the synthesis. 

Unfortunately, the experimental techniques mentioned above do not have spatial resolution, and they cannot detect the proposed patchy distribution of ligands predicted by MD simulations. This limitation motivated the present study.

As in other examples of patchy NCs, the main challenge to obtain a detailed characterization of the patches comes from the nanometric size and location of these patches over the NC surface [1,11,12,13]. Hence, the development of methods providing a direct and reliable picture of the patches of NCs and their nature is of fundamental importance for a reproducible and rational synthesis of patchy nanocrystals. In the above-discussed example [7], the small size of the LnF_3_ particles (<10 nm) impeded a direct characterization of the charges of the individual patches. In this work, we present a strategy designed to overcome this limitation, providing a real-space characterization of LnF_3_ patchy nanocrystals and, in particular, their local electrical charge, morphology, and dimensions.

We divided the work in two parts. First, we present the details of improved synthetic methods of patchy LnF_3_ NCs capable of obtaining a full control over particle size. After exploring diverse strategies, a method for obtaining a tunable range of NC sizes from 5 to ~15 nm is presented. This step is essential to obtain NCs of optimal size with large enough patches suitable for direct characterization by microscopy techniques. Indeed, control of NCs size is a highly desirable feature also for NCs applications in demanding fields where size restrictions are decisive factors (e.g., medical applications) [14,15,16,17]. In the second part, we provide a thorough characterization of optimal size NCs via high-resolution transmission electron microscopy (HRTEM) and Kelvin probe force microscopy (KPFM). We explicitly show the presence of patches with opposite charges at the surface of the nanoparticles.

As far as we know, this is the first reported instance of a complete visualization of these patchy systems, in which the patches themselves and their charge are unveiled.

## 2. Materials and Methods

### 2.1. Materials

Lanthanum(III) acetate hydrate 99.9%, cerium(III) acetate hydrate 99.9%, citric acid 99%, tetramethylammonium hydroxide 25 wt.% in water, and ammonium fluoride >99.99% were purchased from Sigma-Aldrich (Madrid, Spain). Acetone 99.5% was purchased from Scharlau (Barcelona, Spain). All reagents were used as received without further purification.

### 2.2. Nanocrystal Synthesis

**Coprecipitation method.** In a 50 mL round-bottom flask equipped with a condenser and a magnetic stirrer, citric acid (2.25 mmol) in 16 mL of MilliQ water was neutralized with tetramethylammonium hydroxide (6.75 mmol), followed by the addition of Ln(CH_3_COO)_3_·H_2_O (1.5 mmol). The initial solution was maintained at room temperature, heated until 100 °C or introduced in an ultrasonic bath, and then NH_4_F (4.5 mmol) in 4 mL of MilliQ water was injected dropwise. After 2 h of reaction, the final mixture was allowed to reach room temperature depending on the conditions. LnF_3_ particles were separated from the reaction medium by the addition of 10 mL of acetone, followed by centrifugation at 10,000 rpm for 20 min. Separated NCs were redispersed in 20 mL of MilliQ water or methanol, forming stable colloidal dispersions.

**Hydrothermal post-treatment.** Final washed colloidal suspensions in water were added into a Teflon vessel, and then the reactor was sealed; with vigorous stirring, the solution was heated up at 170 or 210 °C for 2 h. Final solutions were washed with acetone at 10,000 rpm for 20 min. Separated NCs were redispersed in 20 mL of MilliQ water or methanol, forming stable colloidal dispersions.

**Hydrothermal method.** In a Teflon vessel, citric acid (2.25 mmol) in 16 mL of MilliQ water was neutralized with tetramethylammonium hydroxide (6.75 mmol), followed by the addition of Ln(CH_3_COO)_3_·H_2_O (1.5 mmol). Then, NH_4_F (4.5 mmol) in 4 mL of MilliQ water was injected dropwise. After the addition, reactor was sealed and heated up at 210 °C. After 2 h of reaction, the final mixture was allowed to reach room temperature. LnF_3_ particles were separated from the reaction medium by the addition of 10 mL of acetone, followed by centrifugation at 10,000 rpm for 20 min. Separated NCs were redispersed in 20 mL of MilliQ water or methanol, forming stable colloidal dispersions.

**Microwave reaction.** In a Teflon vessel, citric acid (2.25 mmol) in 16 mL of MilliQ water was neutralized with tetramethylammonium hydroxide (6.75 mmol), followed by the addition of Ln(CH_3_COO)_3_·H_2_O (1.5 mmol). Then, NH_4_F (4.5 mmol) in 4 mL of MilliQ water was injected dropwise. After the addition, the vessel was introduced in a microwave (MW) to be heated up at 200 °C. After 20 min of reaction, LnF_3_ particles were separated from the reaction medium by the addition of 10 mL of acetone, followed by centrifugation at 10,000 rpm for 20 min. Separated NCs were redispersed in 20 mL of MilliQ water or methanol, forming stable colloidal dispersions.

### 2.3. Characterization Techniques

X-ray powder diffraction (XRD) patterns of the samples were recorded using a Phillips XPert diffractometer equipped with a two circle diffractometers and Cu tube (Malvern Panalytical Ltd., Malvern, UK). Transmission electron microscopy (TEM) micrographs were obtained on a 120 kV JEOL 1210 TEM (JEOL, Freising, Germany), with a resolution point of 3.2 Å. High-Resolution transmission electron microscopy (HRTEM) micrographs were obtained on a 200 kV JEOL 2011 TEM (JEOL, Freising, Germany), with a resolution point of 1.8 Å at 200 kV. Samples for TEM analysis were prepared by spreading a drop of as-prepared NCs diluted dispersion on amorphous carbon-coated grids and then dried in air. All images have been obtained at Servei de Microscòpia of UAB. A microwave (MW) oven of Milestone model FlexiWAVE (Milestone Srl, Bérgamo, Italy) was used to heat up samples at 200 °C. SEM analyses were performed through the Electron Microscopy Service at ICMAB using the QUANTA FEI 200 FEG-ESEM (Hillsboro, OR, USA) with a resolution of 0.8 nm at 30 kV in high vacuum. Atomic force microscopy (AFM) measurements were carried out at room temperature and ambient conditions using the commercial head and control unit from Nanotec Electrónica (Madrid, Spain). Kelvin probe force microscopy (KPFM) was used in the frequency modulation mode (FM-KPFM), in which the tip is excited by an ac voltage (~0.5 V) at a frequency (f_AC_ ~0.7 kHz), while a feedback loop adjusts the DC bias needed to nullify the frequency shift (Δf) at f_AC_. When scanning over a surface, this methodology provides a surface potential (SP) image, which is directly correlated to the local effective work function (φ) and/or to the distribution of electrostatic charges. In our setup, the voltage is applied to the tip such that a higher SP yields a lower φ. The SP and topographic maps are obtained simultaneously, in a single pass, and confirmed in the lift mode, where the tip scans some tens of nm above the surface. This procedure ensures no crosstalk between signals, while it permits establishing direct correspondence between morphology and electrostatic properties. CrPt-coated Si tips mounted in cantilevers with a nominal spring constant of k ≈ 3 N·m^−1^ (BudgetSensors, Sofia, Bulgaria) were used. Water-dispersed NCs were deposited by drop-casting on commercial Au-coated mica substrates (Georg Albert PVD, Silz, Germany) to obtain well-spread distributions of particles all over the sample surface. These substrates guarantee atomically flat (111) oriented terraces of typically several hundreds of nanometers across separated by monoatomic steps (see Figure S1). Different surface locations were measured for each sample containing NCs using the same tip for at least one set of experiments. All data were analyzed with the WSxM freeware (Version 5.0 Develop 9.4, Madrid, Spain) [18].

## 3. Results and Discussion

### 3.1. Tuning the Size of Patchy LnF_3_ NCs

Among the different methods employed to synthesize LaF_3_ NCs with a desired (tunable) size, the first selected approach is an extension of the aqueous coprecipitation method (see Section 2 for details) considered in our previous work [7]. The relatively small NCs obtained from the coprecipitation method are enlarged in size by subsequently applying an appropriate treatment (post-treatment).

**Figure 2:**
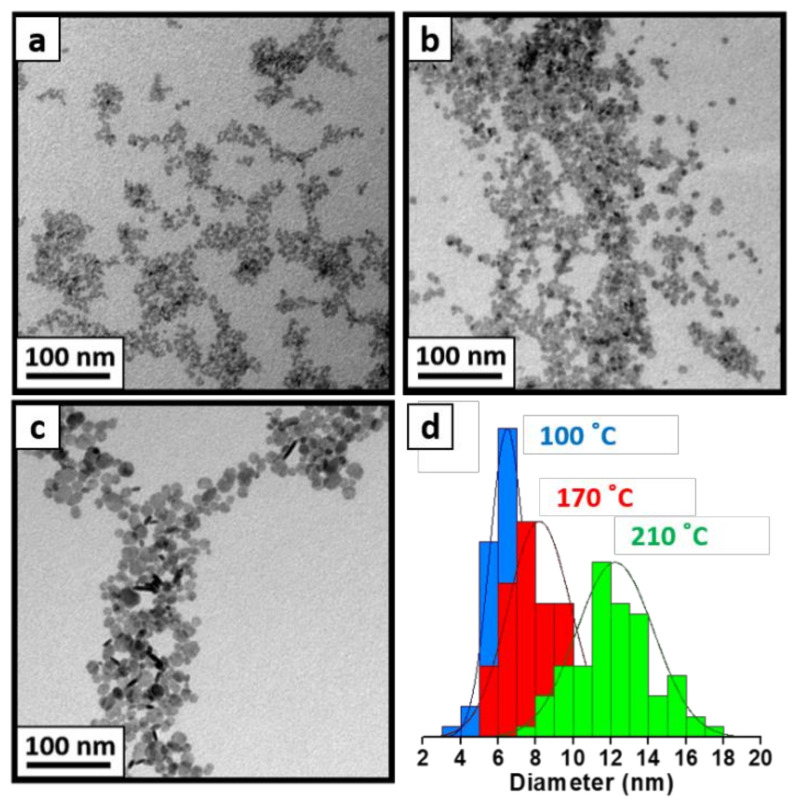
TEM images of as-synthesized LaF_3_ patchy NCs via coprecipitation method at 100 °C (**a**) and after a hydrothermal post-treatment at 170 °C (**b**) and 210 °C (**c**). TEM histograms (**d**) of three images are also shown to corroborate their increasing size: as-synthesized LaF_3_ NCs (blue), LaF_3_ after hydrothermal post-treatment at 170 °C (red), and LaF_3_ after a hydrothermal post-treatment at 210 °C for two additional hours (green).

At room temperature, the aqueous coprecipitation method produces hexagonal crystalline NCs, ~5 nm in size, with or without ultrasonic bath activation (see TEM images and X-ray diffraction patterns in Figures S2 and S3). At 100 °C, the same method leads to larger hexagonal crystalline NCs with a size of ~7 nm (see Figure 2a,d). In order to further increase the NC size, we apply a hydrothermal treatment to the NCs obtained at 100 °C. As illustrated in Figure 2, by using 170 °C and 210 °C for the subsequent enlargement treatment, the final achieved sizes become ~8 nm and ~12 nm, respectively. These results are summarized in Table 1. Noticeably, the enlargement of the LaF_3_ NCs from 7 to 12 nm is accompanied by a better-defined hexagonal shape, i.e., the NCs exhibit improved faceting.

To gain insight into the effect of the post-treatment temperature used, we also employed direct hydrothermal and microwave reactions at similarly high temperatures. In both cases, hexagonal-faceted LaF_3_ NCs with a size of 13–16 nm (Figures S4 and S6) were obtained, with the characteristic crystalline structure (see XRD data in Figures S5 and S7). These results, also summarized in Table 1, indicate that the synthetic methods presented here enable obtaining LaF_3_ NCs with a high control over the size in the range of 5–16 nm, making them suitable for the synthesis of NCs for application where the size is a critical factor. The synthetic approximation discussed here has also been applied to produce other LnF_3_ NCs. Table 1 and Figures S4–S8 include the results for hexagonal crystalline CeF_3_ NCs with sizes ranging from 8 to 14 nm [7].

In summary, LaF_3_ and CeF_3_ NCs of different sizes can be synthesized using diverse aqueous methodologies (Table 1), all of them using the same precursors and with the same resulting crystalline structure.

As shown schematically in Figure 1a, all LnF_3_ NCs reported in Table 1 are expected to be stabilized by coordination of surface atoms with ligands initially present in the synthesis medium [7]. In our case, the synthesis medium contained citrate and acetate anions and ammonium and tetramethylammonium cations (see Section 2). According to the mechanism described in Figure 1, we predict that the hexagonal surfaces of LnF_3_ NCs are stabilized by citrate and acetate anions coordinated to La or Ce, acquiring a negative charge, and the rectangular surfaces are stabilized by ammonium cations, acquiring a positive charge (Figure 2a). This prediction is directly tested in the next subsection.

### 3.2. Full Imaging of Patchy LnF_3_ Nanocrystals

In this subsection, we describe the characterization of patchy NCs, including the imaging of their facets and the identification of differently charged patches. We perform this characterization for the case of the CeF_3_ NCs synthesized via hydrothermal treatment at 210 °C (see Table 1) since they have an optimal size for the techniques employed here.

**Figure 3:**
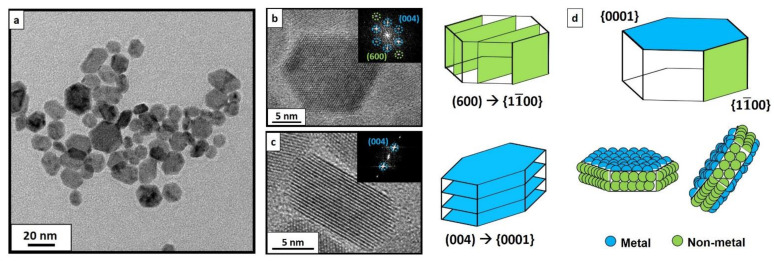
TEM image of CeF_3_ NCs synthesized via hydrothermal method at 210 °C (**a**). Well faceted-NCs could be observed tilted in several directions, randomly oriented. HRTEM images of as-synthesized CeF_3_ NCs via hydrothermal treatment at 210 °C with their corresponding faceting in {1$\bar{1}$00} (**b**) and in {0001} (**c**) planes. Deduced experimental faceting, as well as the exposed elements in each facet (**d**).

The particle faceting is clearly seen in the representative HRTEM images of the oriented CeF_3_ NCs (Figure 3). In view of the hexagonal face shown in Figure 3b, (004) and (600) planes are correspondingly indexed. This particular configuration is explained thanks to the specific orientations adopted by the NC (flat in Figure 3b, on-edge in Figure 3c) that allow visualizing both facets. However, a closer look at the NC when oriented through their rectangular face (Figure 3c) reveals that only the (004) oriented plane is clearly distinguished, meaning that the hexagonal exposed face is {0001}. Thus, the rectangular planes observed in Figure 3b must have (600) orientation with an exposed face of the {1$\bar{1}$00} family.

As commented above, fluorine-rich {1$\bar{1}$00} patches would be stabilized by cations, generating a positive global charge. In contrast, {0001} hexagonal facets were metal-terminated and, therefore, the attachment of anions onto them would lead to a final local negative charge. To identify the selectively charged patches, we consider how the charges are distributed and where they are located (Figure 1a). Hexagonal {0001} faces exposed in flat lying NCs are relatively large and, therefore, easily detected via TEM (see also Figure S4). In contrast, visualizing {1$\bar{1}$00} planes, exhibiting smaller lateral extension, is challenging unless a high number of particles are lying in the on-edge configuration (i.e., exposing this facet as in the model of Figure 1a).

**Figure 4:**
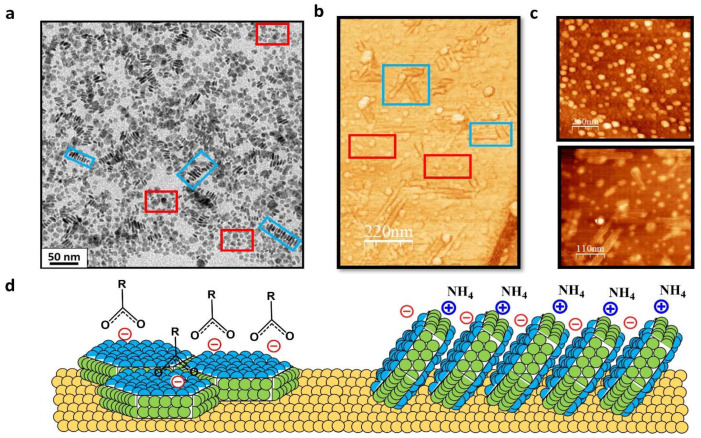
(**a**) TEM image of high-concentrated CeF_3_ NCs synthesized via hydrothermal treatment at 210 °C. Here, the NCs can be observed in a planar (lying flat—red squares) distribution and in a perpendicular (on-edge—blue squares) configuration. In the latter, the interaction between NCs leads to the formation of rod-like assemblies. (**b**) AFM error signal image enhancing morphological details of an area containing planar and rod-like configurations of CeF_3_ NCs deposited by drop-casting on Au(111) in both orientations, as detailed in Figure 4a. (**c**) Topographic AFM images of two different areas: (top) area (1300 nm × 1300 nm) containing NCs in the planar distribution and (bottom) area (550 nm × 550 nm) showing the rod-like interaction. Total color scales are Δz = 10 nm. (**d**) Schematic models for the two NCs configurations found in the TEM and AFM images and the respective observed distributions: (left) planar NCs and (right) rod-like particles. We also indicate the adsorbed ligands and their anionic or cationic character.

Due to the adequate ratio between NCs adopting each type of configuration (i.e., exposing one or the other plane), the facet-dependent behavior characteristic of LnF_3_ NCs, can be studied here. As seen by TEM (Figure 4a), the preparation of highly concentrated solutions (~150 mM) of these NCs led to two types of NC organizations: (i) monodisperse NCs lying flat on the grid and exposing their {0001} planes, highlighted with red squares, and (ii) rod-like assemblies of NCs exposing {1$\bar{1}$00} planes, highlighted with blue squares. These structures are only formed at such high concentrations in solution, allowing the possible creation of analogous ionic bridge interactions [19] between NCs, permitting directional self-assembly through {0001} planes. Indeed, the unique on-surface rod-like NCs organization, in which the {1$\bar{1}$00} planes are exposed, matches the commented need for having a high number of particles in a perpendicular position to access these facets by imaging techniques.

Samples consisting of atomically flat Au (111) films, in which the water dispersion of the NCs was deposited by drop-casting (see Section 2) were measured by AFM over areas previously localized by SEM (Figure S9). Figure 4b shows a general view of the most observed morphological details. As expected from a planar configuration of the platelet-like individual NCs with the {0001} facet parallel to the Au substrate surface, randomly distributed particles with heights of 3–10 nm and apparent width from 10 nm to 60 nm depending on tip conditions (Figure 4c, top) were found in the vast majority of the inspected areas. Albeit in a small amount all over the surface, some baton-shaped structures (100–200 nm long) were also found (Figure 4c, bottom), ascribed to the rod-like assemblies observed by TEM. Resulting from the attachment of the interacting hexagonal facets, in this configuration, the {0001} planes lie out of the surface plane. As observed in Figure 4b, considerable amounts of rods were found in surface locations close to surface steps. Remarkably, all rods appear oriented along three directions 60° rotated between each other (Figure 4b,c bottom), indicating the influence of the underlying Au (111) symmetry. Statistical analysis using images taken in different areas indicates that these elongated structures are typically 30–40 nm wide, in agreement with the NC diameter measured in the planar configuration, but they only have an apparent height of ~2–4 nm. Given the excellent vertical resolution of the topographic data (see Au (111) monoatomic steps in Figure S1), this small out-of-plane dimension suggests that the NCs must be tilted with respect to the normal surface (see models of both configurations in Figure 4d).

**Figure 5:**
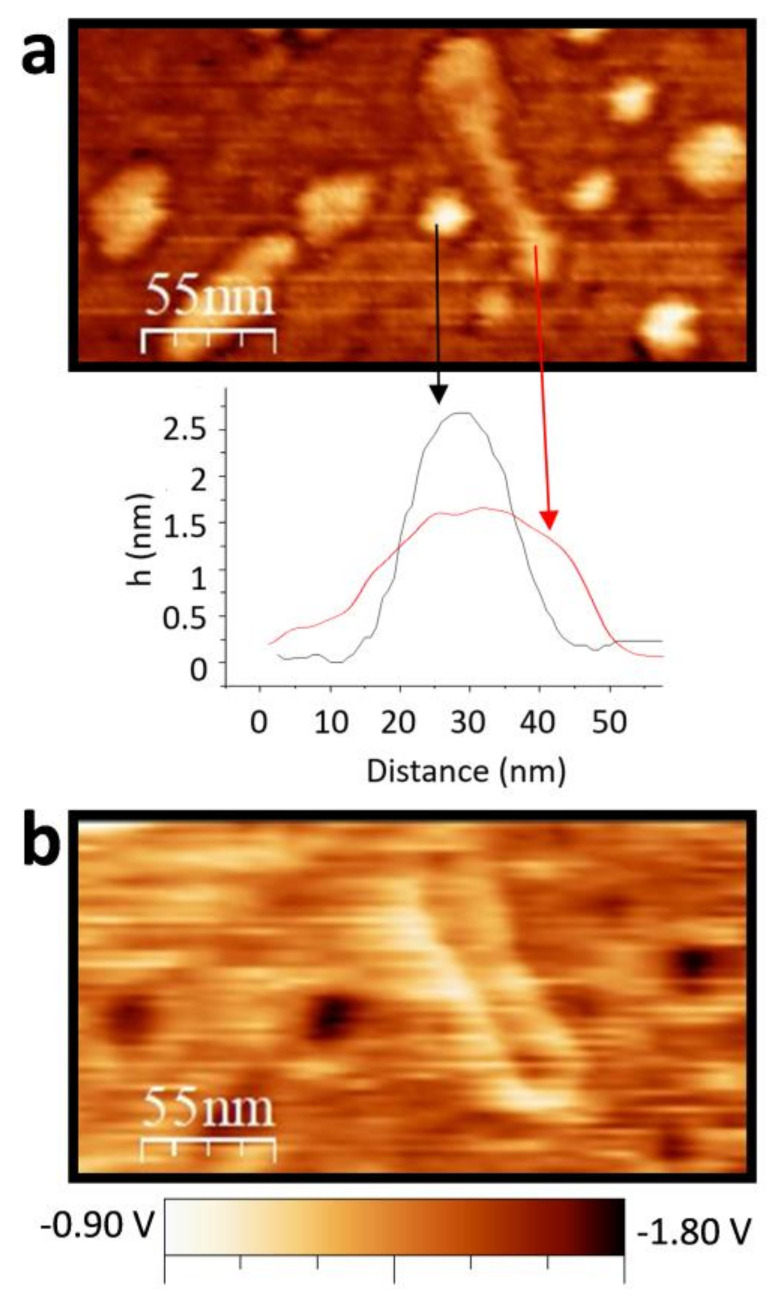
(**a**) Topographic image and line profiles of typical individual NCs (black) and across the shorter dimension of the rod-like assemblies of inclined NCs. (**b**) Simultaneous acquired surface potential map by KPFM. Images size is 275 nm × 145 nm and the color scales are Δz = 10 nm (**a**) and ΔSP = 900 mV (**b**) from white to black. The surface potential is lower on the exposed {0001} facets than on the surface of the rod (see experimental details and complementary analysis in Figure S10).

The contrast in the KPFM maps, arising from the compensation of electrostatic forces at the local level, is commonly attributed to local differences in work function [20], charges, or electrical dipoles [21]. Therefore, KPFM measurements permit obtaining electrostatic surface potential (SP) with local variations at the nanoscale, and they have demonstrated their capability to provide information about surface charges in nanocrystals [22]. Because topography and spatially resolved SP maps are recorded simultaneously, the direct comparison between data in Figure 5 provides a correlation between the NCs organization observed in the topographical image (a) with the corresponding surface potential signal, which results more negative (darker) for the planar NCs and more positive (brighter) for the long assemblies (b). In terms of SP quantification, it is worth mentioning here several facts. On the one hand, the absolute nominal charge of each facet cannot be accurately evaluated by this method because of the unknown extent of (i) charge screening by the metallic support, and/or (ii) variations in charge state with environmental conditions due to their hydrophobic/hydrophilic character. On the other hand, the inclination of the NCs in the rods implies that, in addition to the {1$\bar{1}$00} facets, there is a contribution of the exposed section of the {0001} planes (see alternate + and − signs in Figure 4d, right), likely provoking altered magnitude of the probed local surface potential. Despite all these considerations, the corresponding SP image (Figure 5b) shows a clear contrast between both types of structures. The surface potential measured on the planar individual NCs (showing {0001} planes) is, on average, about 280 mV more negative than for the assemblies of inclined NCs, where the {1$\bar{1}$00} planes dominate (see Figure S10). These results nicely confirm the predictions shown in Figure 1 based on all-atom MD simulations.

These findings are in agreement with the expected dual faceted-charge behavior: a positive charge distribution for the rods due to the interaction of the tip with the nonmetal planes (Figure 4d right) and a negative charge distribution in the planar NCs due to the exposed hexagonal metallic plane (Figure 4d left). As far as we know, this is the first experimental evidence via a microscopy technique of the structural and charge patches in these kinds of systems.

## 4. Conclusions

In summary, we reported the tunable-size synthesis of faceted-charge patchy NCs within a range suitable for material science and medical applications (i.e., NCs of a size >10 nm are mandatory for reducing toxicity in biological systems) [23,24]. Moreover, we provided a microscopic picture of their surface dual properties, hydrophilic/hydrophobic composition, and charged patches. We established a direct comparison at the nanoscale between the electrostatic response of flat-lying single NCs exposing the metal-terminated {0001} plane and that of rod-like structures consisting of tilted NCs with most of their exposed surface corresponding to nonmetal {1$\bar{1}$00} planes. A notable difference in surface potential as measured by KPFM between the two NC organizations evidenced the dual-charge nature of the patchy system. This work established the foundations for real-space visualization of patchy systems, going one step further in the characterization of single NCs and the identification of their diverse functional sites at the nanoscale. Furthermore, this work demonstrated the suitability of KPFM in identifying the presence of patches of opposite charge in nanoparticles if the size of the particles is suitable for the analysis.

## Figures and Tables

**Table 1:** Size of all LaF_3_ and CeF_3_ NCs obtained by TEM histograms. The synthesis methods or post-treatments are indicated as follows: Co-Prec., coprecipitation; Hydro., hydrothermal; MW, microwave; rt, room temperature. * Data obtained from [7] for comparison.

NCs	1st Treatment	2nd Treatment	TEM Size (nm)
LaF_3_	Co-Prec. Rt	-	4.7 ± 1.4
	Co-Prec. rt + sonication	-	4.7 ± 1.0
	Co-Prec. 100 °C	-	7.0 ± 1.0
	Co-Prec. 100 °C	Hydro. 170 °C	8.2 ± 1.7
	Co-Prec. 100 °C	Hydro. 210 °C	12.2 ± 2.1
	Hydro. 210 °C	-	15.7 ± 3.5
	MW 200 °C	-	13.0 ± 3.3
CeF_3_	Co-Prec. 100 °C *	-	7.6 ± 2.3
	Hydro. 210 °C	-	12.9 ± 3.3
	MW 200 °C	-	14.2 ± 3.4

## Data Availability

All data are available in the main manuscript and Supplementary Materials.

## Supplementary Materials

The following are available online at https://www.mdpi.com/article/10.3390/ma15041455/s1: Figure S1: AFM topographic images of Au(111) on mica at two different magnifications. Terraces of several hundred nanometers in width are separated by monoatomic steps (2.35Å) as corresponds to the distance between (111) planes. This vertical distance is used for accurate Z calibration of the images; title; Figure S2: TEM images of LaF3 NCs synthesised at (A) room temperature and (B) room temperature in an ultrasonic batch during 2 h; Figure S3: X-ray diffraction patterns of LaF3 NCs obtained at (A) room temperature and (B) under ultrasonic bath, both of them using the co-precipitation method; Figure S4: TEM images of LaF3 (A) and CeF3 (B) patchy NCs via hydrothermal reaction at 210 °C for 2 h and LaF3; Figure S5: X-ray diffraction patterns of (A) LaF3 and (B) CeF3 NCs obtained at 210 °C in a hydrothermal synthesis during 2 h; Figure S6: TEM images of LaF3 (A) and CeF3 (B) patchy NCs via microwave reaction at 200 °C for 20 min; Figure S7: X-ray diffraction patterns of (A) LaF3 and (B) CeF3 NCs obtained at 200 °C in a microwave synthesis during 20 minutes; Figure S8: TEM Histograms corresponding to the NCs showed in this paper and summarized in Table 1. (A) LaF3 synthesised at room temperature with and without ultrasonic bath. (B) LaF3 and (C) CeF3 synthesised via high-thermal methods; Figure S9: Scanning Electron microscopy (SEM) image and Electron dispersive X-ray (EDX) of the CeF3 NCs to ensure their presence before AFM technique; Figure S10: (A) Topographical and (B) SP images obtained by KPFM of an area where planar NCs and piles were found with their corresponding numerical plots: (C) hexagonal {0001} faces and (D) piles showing the exposed {1$\bar{1}$00} planes. The areas of the topographical and SP profiles of NCs (green) and pile (blue) are marked in Figure S10a.

## References

[1] Choueiri R.M., Galati E., Thérien-Aubin H., Klinkova A., Larin E.M., Querejeta-Fernández A., Han L., Xin H.L., Gang O., Zhulina E.B. (2016). Surface patterning of nanoparticles with polymer patches. Nature.

[2] Velev O.D. (2013). Curvature makes a difference. Nat. Nanotechnol..

[3] Zhang Z., Glotzer S.C. (2004). Self-Assembly of Patchy Particles. Nano Lett..

[4] Fan W., Liu L., Zhao H. (2017). Co-assembly of Patchy Polymeric Micelles and Protein Molecules. Angew. Chem. Int. Ed. Engl..

[5] Pons-Siepermann I.C., Glotzer S.C. (2012). Electromechanical Actuator with Controllable Motion, Fast Response Rate, and High-Frequency Resonance Based on Graphene and Polydiacetylene. ACS Nano.

[6] Harper E.S., Van Anders G., Glotzer S.C. (2019). The entropic bond in colloidal crystals. Proc. Natl. Acad. Sci. USA.

[7] Martínez-Esaín J., Puig T., Obradors X., Ros J., Yáñez R., Faraudo J., Ricart S. (2018). Faceted-Charge Patchy LnF 3 Nanocrystals with a Selective Solvent Interaction. Angew. Chem. Int. Ed. Engl..

[8] Owen J. (2015). Nanocrystal structure. The coordination chemistry of nanocrystal surfaces. Science.

[9] Boles M.A., Ling D., Hyeon T., Talapin D.V. (2016). The surface science of nanocrystals. Nat. Mater..

[10] Humphrey W., Dalke A., Schulten K.J. (1996). VMD: Visual molecular dynamics. J. Mol. Graph..

[11] Luo B., Smith J.W., Wu Z., Kim J., Ou Z., Chen Q. (2017). Polymerization-Like Co-Assembly of Silver Nanoplates and Patchy Spheres. ACS Nano.

[12] Pothorszky S., Zámbó D., Szekrényes D., Hajnal Z., Deák A. (2017). Detecting patchy nanoparticle assembly at the single-particle level. Nanoscale.

[13] See E.M., Peck C.L., Santos W.L., Robinson H.D. (2017). Light-Directed Patchy Particle Fabrication and Assembly from Isotropic Silver Nanoparticles. Langmuir.

[14] Cheng L., Yang K., Li Y., Zeng X., Shao M., Lee S., Liu Z. (2012). Multifunctional nanoparticles for upconversion luminescence/MR multimodal imaging and magnetically targeted photothermal therapy. Biomaterials.

[15] Sun Y., Yu M., Liang S., Zhang Y., Li C., Mou T., Yang W., Zhang X., Li B., Huang C. (2011). Fluorine-18 labeled rare-earth nanoparticles for positron emission tomography (PET) imaging of sentinel lymph node. Biomaterials.

[16] Xia A., Gao Y., Zhou J., Li C., Yang T., Wu D., Wu L., Li F. (2011). Core–shell NaYF4:Yb3+, Tm3+ @FexOy nanocrystals for dual-modality T2-enhanced magnetic resonance and NIR-to-NIR upconversion luminescent imaging of small-animal lymphatic node. Biomaterials.

[17] Xiong L., Shen B., Behera D., Gambhir S.S., Chin F.T., Rao J. (2013). Synthesis of ligand-functionalized water-soluble[18F]YF3 nanoparticles for PET imaging. Nanoscale.

[18] You A., Be M.A.Y., In I. (2007). WSXM: A software for scanning probe microscopy and a tool for nanotechnology. Rev. Sci. Instrum..

[19] Martínez-Esaín J., Faraudo J., Puig T., Obradors X., Ros J., Ricart S., Yáñez R. (2018). Tunable Self-Assembly of YF3 Nanoparticles by Citrate-Mediated Ionic Bridges. J. Am. Chem. Soc..

[20] Sadewasser S., Glatzel T., Rusu M., Lux-Steiner M.C. (2012). High-resolution work function imaging of single grains of semiconductor surfaces. Appl. Phys. Lett..

[21] Barth C., Henry C.R. (2011). Gold nanoclusters on alkali halide surfaces: Charging and tunneling. Appl. Phys. Lett..

[22] Geisel K., Rudov A.A., Potemkin I.I., Richtering W. (2015). Hollow and Core–Shell Microgels at Oil–Water Interfaces: Spreading of Soft Particles Reduces the Compressibility of the Monolayer. Langmuir.

[23] Albanese A., Tang P.S., Chan W.C.W. (2012). The effect of nanoparticle size, shape, and surface chemistry on biological systems. Annu. Rev. Biomed. Eng..

[24] Nel A., Xia T., Mädler L., Li N. (2006). Toxic Potential of Materials at the Nanolevel. Science.

