# Supplementary material for: Real-Space Image of Charged Patches in Tunable-Size Nanocrystals
Source: Materials (Basel). 2022 Feb 15;15(4):1455. doi: 10.3390/ma15041455 (PMC8875652; doi:10.3390/ma15041455)
Supplement: Supplementary file 1 [file materials-15-01455-s001.zip › materials-1501634-supplementary.pdf]

materials

MDPI

Supporting Information

Real-Space Image of Charged Patches in Tuneable Size Nanocrystals

Jordi Martínez-Esaín [1,2], Ana Perez-Rodriguez [2], Jordi Faraudo [2,*], Esther Barrena [2], Ramón Yáñez [1], Carmen Ocal [2] and Susagna Ricart [2,*]

[1] Departament de Quimica, Universitat Autonoma de Barcelona, 08193 Bellaterra, Spain
[2] Institut de Ciencia de Materials de Barcelona (ICMAB-CSIC), 08193 Bellaterra, Spain
[*] Correspondence: jfaraudo@icmab.es (J.F.); ricart@icmab.es (S.R.)

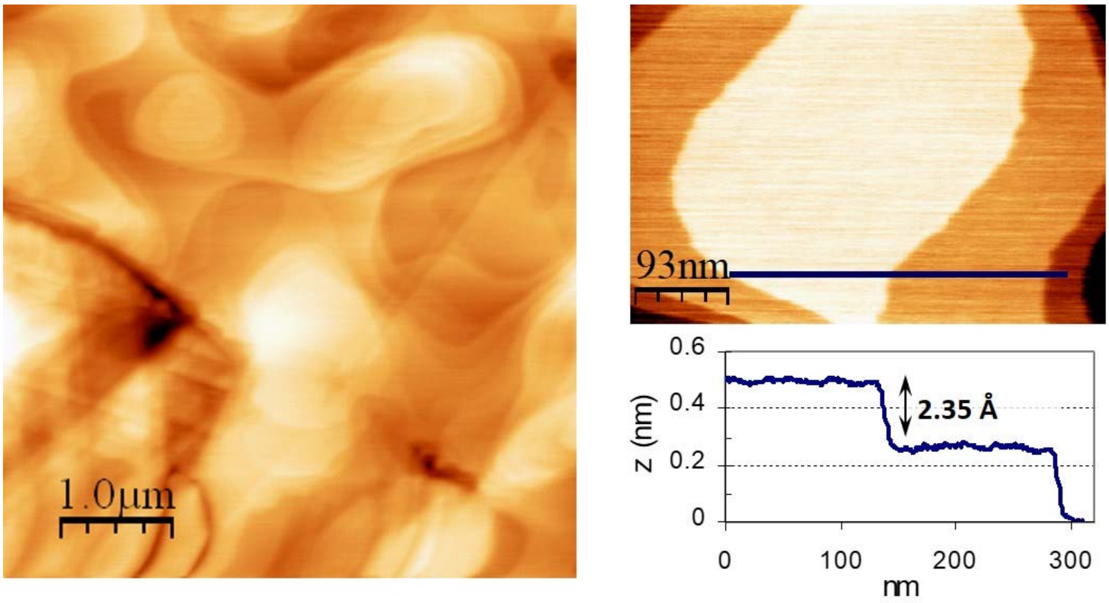

Figure S1. AFM topographic images of Au(111) on mica (substrates (Georg Albert PVD, Silz, Germany) at two different magnifications. Terraces of several hundred nanometers in width are separated by monoatomic steps (2.35 Å) as corresponds to the distance between (111) planes. This vertical distance is used for accurate Z calibration of the images.

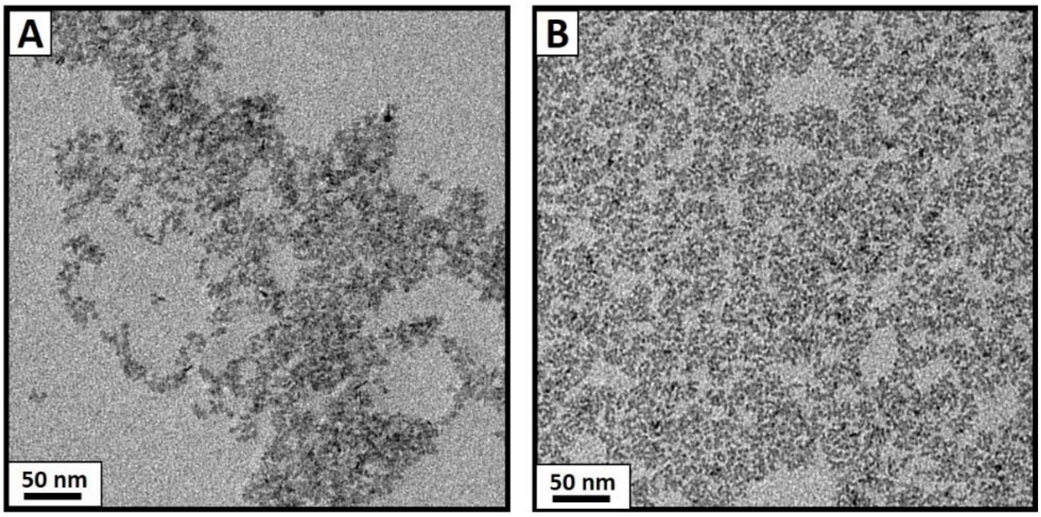

Figure S2. TEM images of LaF_3 NCs synthesised at (**A**) room temperature and (**B**) room temperature in an ultrasonic batch during 2 hours.

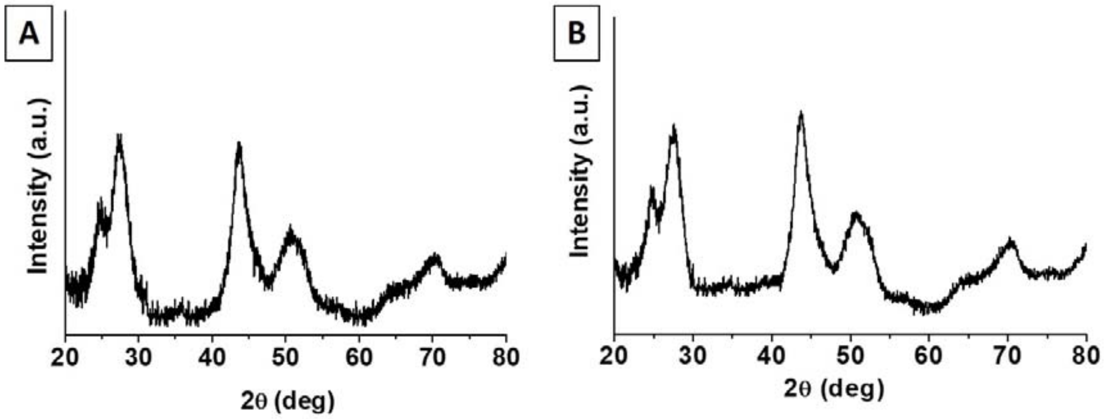

Figure S3. X-ray diffraction patterns of LaF_3 NCs obtained at (**A**) room temperature and (**B**) under ultrasonic bath, both of them using the co-precipitation method.

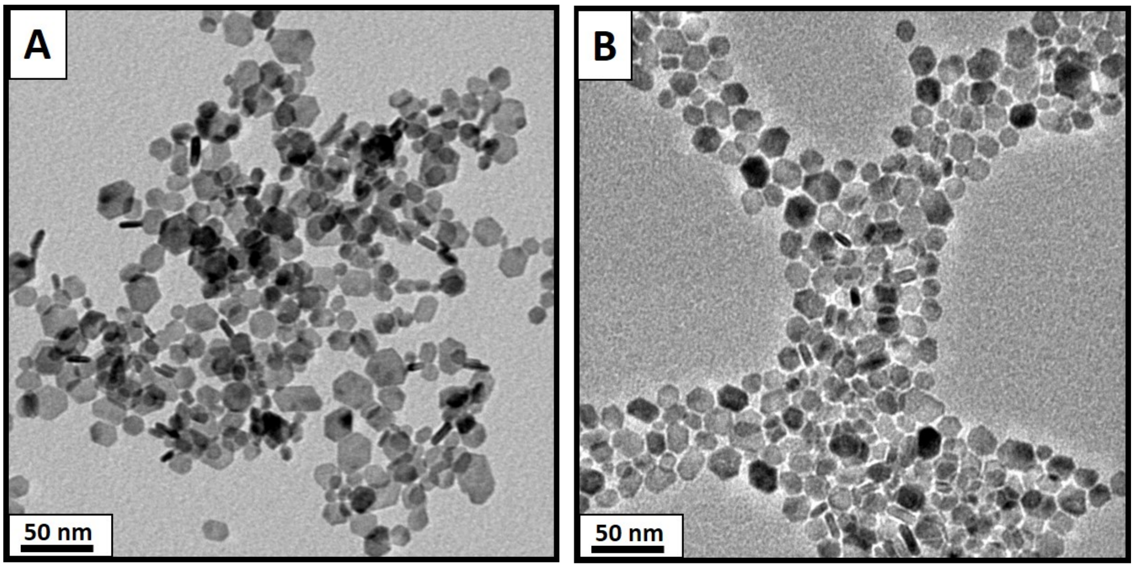

Figure S4. TEM images of LaF_3 (**A**) and CeF_3 (**B**) patchy NCs via hydrothermal reaction at 210 °C for 2 hours and LaF_3.

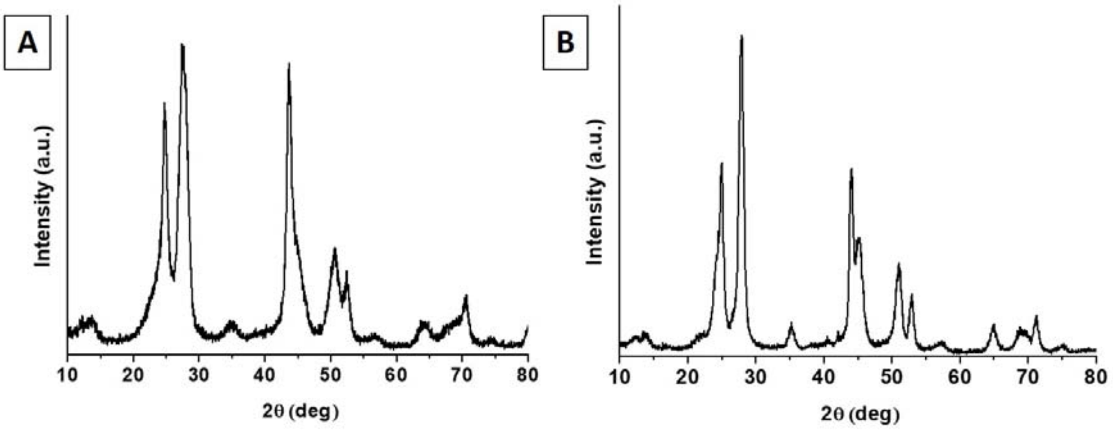

Figure S5. X-ray diffraction patterns of (**A**) LaF_3 and (**B**) CeF_3 NCs obtained at 210 °C in a hydrothermal synthesis during 2 hours.

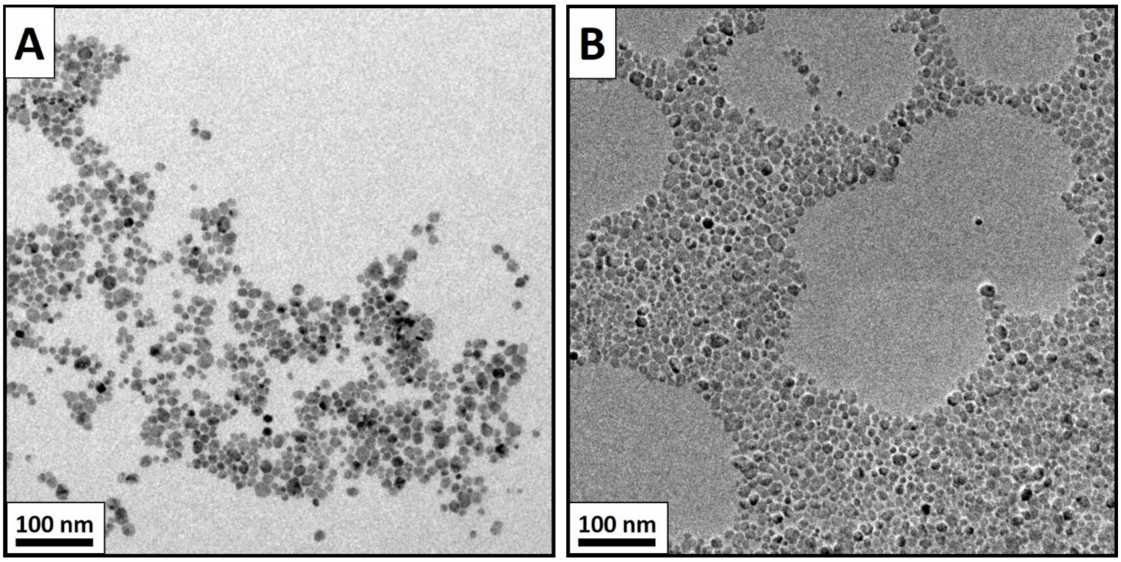

Figure S6. TEM images of LaF_3 (**A**) and CeF_3 (**B**) patchy NCs via microwave reaction at 200 °C for 20 min.

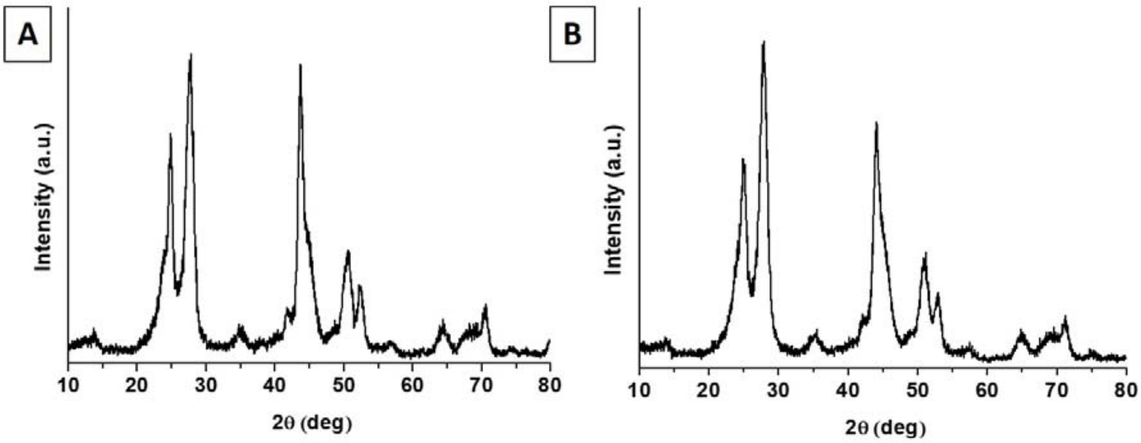

Figure S7. X-ray diffraction patterns of (**A**) LaF_3 and (**B**) CeF_3 NCs obtained at 200 °C in a microwave synthesis during 20 minutes.

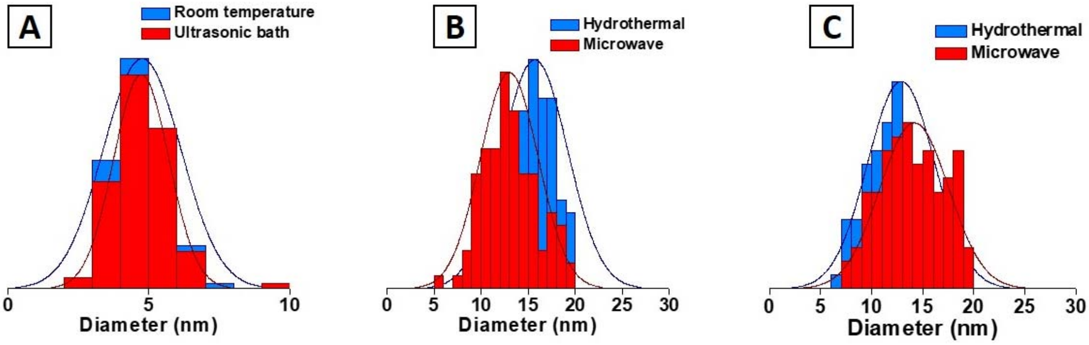

Figure S8. TEM Histograms corresponding to the NCs showed in this paper and summarized in Table 1. (**A**) LaF_3 synthesised at room temperature with and without ultrasonic bath. (**B**) LaF_3 and (**C**) CeF_3 synthesised via high-thermal methods.

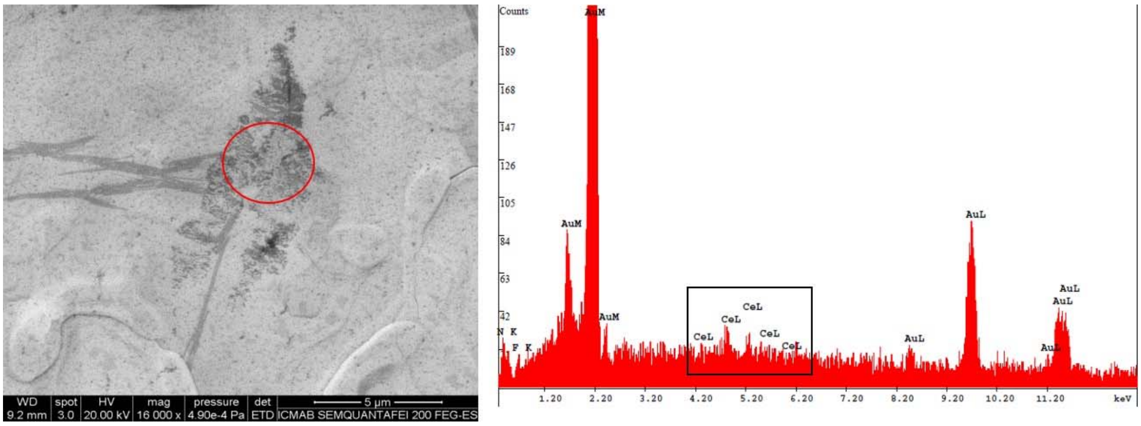

Figure S9. Scanning Electron microscopy (SEM) image and Electron dispersive X-ray (EDX) of the CeF_3 NCs to ensure their presence before AFM technique.

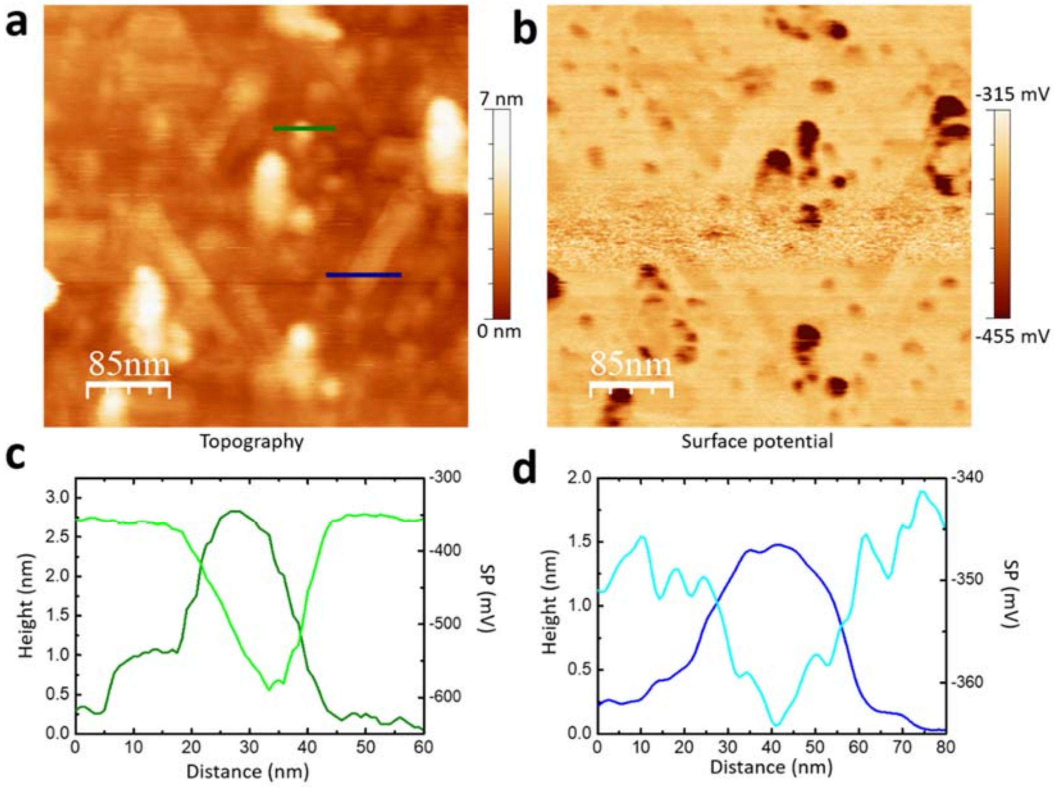

Figure S10. (**a**) Topographical and (**b**) SP images obtained by KPFM of an area where planar NCs and piles were found with their corresponding numerical plots: (**c**) hexagonal {0001} faces and (**d**) piles showing the exposed {1$\bar{1}$00} planes. The areas of the topographical and SP profiles of NCs (green) and pile (blue) are marked in Figure S10a.
